# miR-134 Modulates the Proliferation of Human Cardiomyocyte Progenitor Cells by Targeting *Meis2*

**DOI:** 10.3390/ijms161025199

**Published:** 2015-10-23

**Authors:** Ya-Han Wu, Hong Zhao, Li-Ping Zhou, Chun-Xia Zhao, Yu-Fei Wu, Li-Xiao Zhen, Jun Li, Dong-Xia Ge, Liang Xu, Li Lin, Yi Liu, Dan-Dan Liang, Yi-Han Chen

**Affiliations:** 1 Key Laboratory of Arrhythmias of the Ministry of Education of China, East Hospital, Tongji University School of Medicine, Shanghai 200120, China; E-Mails: wuyahan_qirmai@163.com (Y.-H.W.); applezhou1991@163.com (L.-P.Z.); youyou131205@hotmail.com (C.-X.Z.); 111yufei_wu@tongji.edu.cn (Y.-F.W.); zlxiao56@163.com (L.-X.Z.); junli@tongji.edu.cn (J.L.); gedongx@163.com (D.-X.G.); xuliang_east@126.com (L.X.); lilincn1@163.com (L.L.); yiliu@tongji.edu.cn (Y.L.); 2 Research Center for Translational Medicine, East Hospital, Tongji University School of Medicine, Shanghai 200120, China; 3 Institute of Medical Genetics, Tongji University, Shanghai 200092, China; 4 Department of Cardiology, East Hospital, Tongji University School of Medicine, Shanghai 200120, China; 5 Department of Pediatrics, Tongji Hospital, Tongji University, Shanghai 200120, China; E-Mail: hongzhao_tj@sohu.com; 6 Department of Pathology and Pathophysiology, Tongji University School of Medicine, Shanghai 200092, China

**Keywords:** hCMPCs, miR-134, proliferation, *Meis2*

## Abstract

Cardiomyocyte progenitor cells play essential roles in early heart development, which requires highly controlled cellular organization. microRNAs (miRs) are involved in various cell behaviors by post-transcriptional regulation of target genes. However, the roles of miRNAs in human cardiomyocyte progenitor cells (hCMPCs) remain to be elucidated. Our previous study showed that miR-134 was significantly downregulated in heart tissue suffering from congenital heart disease, underlying the potential role of miR-134 in cardiogenesis. In the present work, we showed that the upregulation of miR-134 reduced the proliferation of hCMPCs, as determined by EdU assay and Ki-67 immunostaining, while the inhibition of miR-134 exhibited an opposite effect. Both up- and downregulation of miR-134 expression altered the transcriptional level of cell-cycle genes. We identified *Meis2* as the target of miR-134 in the regulation of hCMPC proliferation through bioinformatic prediction, luciferase reporter assay and western blot. The over-expression of *Meis2* mitigated the effect of miR-134 on hCMPC proliferation. Moreover, miR-134 did not change the degree of hCMPC differentiation into cardiomyocytes in our model, suggesting that miR-134 is not required in this process. These findings reveal an essential role for miR-134 in cardiomyocyte progenitor cell biology and provide new insights into the physiology and pathology of cardiogenesis.

## 1. Introduction

Cardiomyocyte progenitor cells arise in the mesodermal layer at the embryonic stage and contribute to the final shape of the heart with its morphologically distinct compartments [1]. Abnormal progenitor cell behaviors during cardiogenesis, including their proliferation and differentiation, leads to cardiac malformations [2]. In addition in to the embryonic heart, resident cardiac progenitor cells have also been identified in adults [3]. These cells, which have shown the potential to form cardiomyocytes, smooth muscle cells, and endothelial cells *in vitro* and *in vivo*, could potentially be used as a source for cardiac repair [4]. Insight into the regulation of cardiomyocyte progenitor cells is crucial for the recognition of many cardiogenic processes. The functional role of CMPCs has been tested in ischemic murine heart, in which the transplantation of CMPCs improved heart function [4]. Human cardiomyocyte progenitor cells (hCMPCs) were isolated from fetal hearts or adult biopsies, could be induced to differentiate into cardiomyocytes and are independent of any feeder cells in the culture, which provide a model to study cardiac progenitor cells or cardiomyocyte function and signaling [5].

MicroRNAs (miRs) are a large class of small, conserved, noncoding RNAs, which primarily function post-transcriptionally by base-pairing with the 3′ untranslated region (UTR) of target mRNAs [6]. MiRs have been demonstrated to participate in a broad range of cardiac physiological and pathophysiological processes. Identification of miRs in different heart diseases has led to the exploration of regulatory roles for these small RNAs during cardiomyocyte differentiation, cell cycle, and cardiac hypertrophy [7]. Several miRNAs have been functionally characterized in hCMPCs. miR-1 and miR-499 have been reported to reduce the proliferation rate and enhance differentiation into cardiomyocytes in hCMPCs [8]. Our previous study demonstrated that miR-204 was required for the proliferation and differentiation of hCMPCs [9]. Recently, we have identified a group of differentially expressed miRNAs by an miRNA array analysis in heart tissue between Tetralogy of Fallot (TOF) patients and healthy individuals [10]. MiR-134 was significantly downregulated in TOF patients, suggesting the potential role of miR-134 in the heart development and proliferation/differentiation of hCMPCs.

Here, we sought to explore the effects of miR-134 on hCMPCs and reveal its mechanism of function. We showed that miR-134 repressed hCMPC proliferation and did not influence the differentiation of hCMPCs toward cardiomyocytes. We also identified *Meis2* as the target of miR-134. *Meis2* overexpression rescues the phenotype of miR-134 in hCMPCs.

## 2. Results

### 2.1. miR-134 Modulates hCMPCs Proliferation

**Figure 1:**
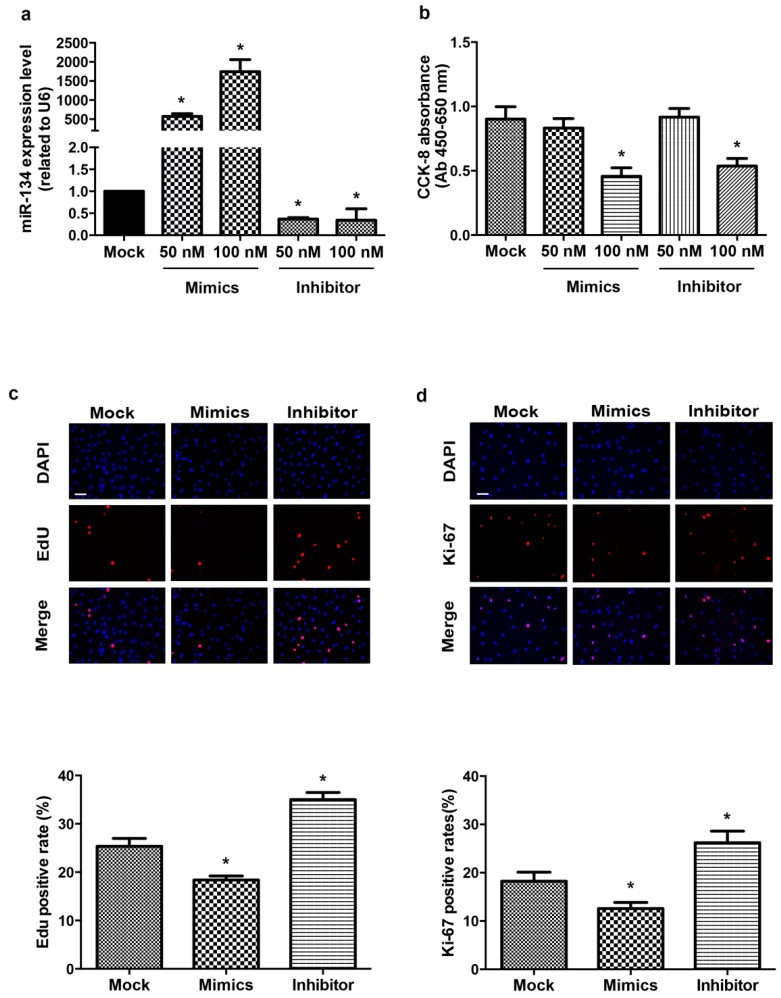
miR-134 regulates the proliferation of hCMPCs. (**a**) Quantification of miR-134 expression level after the transfection of different concentrations (50 and 100 nM) of miR-134 mimics or inhibitor; (**b**) Effect of miR-134 mimics or inhibitor on hCMPC viability, as detected using the CCK-8 assay; (**c**) Representative images and statistical data of hCMPCs transfected with mock, miR-134 mimics or inhibitor stained with DAPI and EdU; (**d**) Representative images and statistical data of hCMPCs transfected with mock, miR-134 mimics or inhibitor stained with DAPI and Ki-67. Bar = 75 μm. * *p* < 0.05, Data were from five independent experiments.

To examine the physiological expression level of miR-134, we performed the absolute quantification for miR-134 in proliferating hCMPCs (Figure S1). To investigate whether miR-134 affects the proliferation of hCMPCs, we first evaluated the expression levels of miR-134 and the cytotoxic effects after the transient transfection of different concentrations of miR-134 mimics or inhibitor. The results showed that the miR-134 mimics (50 and 100 nM) significantly increased the expression level of miR-134 compared with the scramble control, while miR-134 inhibitor (50 and 100 nM) decreased the level (Figure 1a). The cell viability was then detected using the CCK-8 assay. As shown in Figure 1b, 100 nM miR-134 mimics or inhibitor decreased hCMPC cell viability, implicating 50 nM as an optimal concentration for our study.

**Figure 2:**
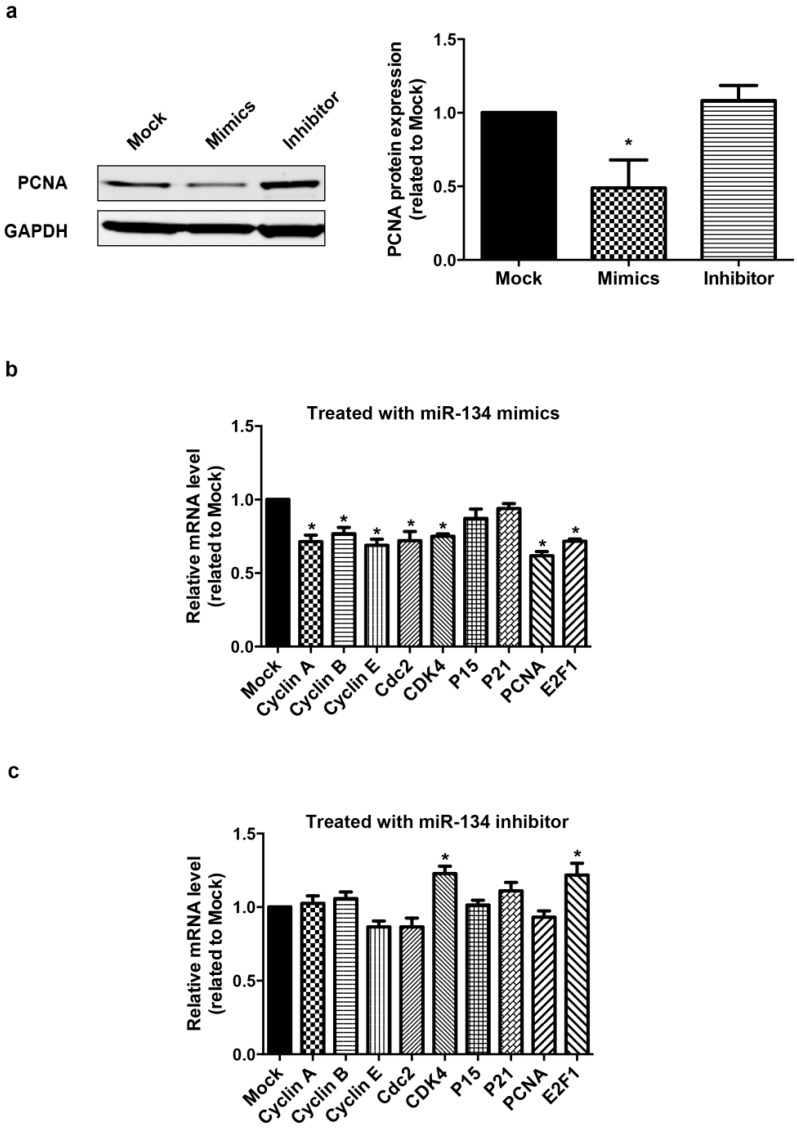
Modulation of miR-134 changes the expression of the cell cycle regulatory genes of hCMPCs. (**a**) PCNA protein is down-regulated in hCMPCs transfected with miR-134 mimics; (**b**) Relative expression of cell cycle regulatory genes in hCMPCs transfected with miR-134 mimics; (**c**) The same set of genes was measured in hCMPCs with the inhibition of miR-134. The expression level of GAPDH was used as the control. * *p* < 0.05. Data were from five independent experiments.

hCMPCs proliferation was determined by an EdU assay, which detects incorporated EdU and directly measures DNA synthesis. The percentage of EdU-positive hCMPCs significantly decreased by 27.6% upon treatment with miR-134 mimics, while the miR-134 inhibitor increased it by 38.1% (Figure 1c). We also detected the expression of Ki-67, which is a nuclear protein associated with cellular proliferation, to assess the proliferation of hCMPCs. Consistent with the EdU assay, Ki-67 immunostaining showed that hCMPC proliferation could be altered by modulating the expression level of miR-134 (Figure 1d). Furthermore, the protein expression of Proliferating Cell Nuclear Antigen (PCNA) in hCMPCs was examined after the transfection of miR-134 mimics or inhibitor. PCNA is a component of the DNA replication fork and is required for DNA synthesis and repair, commonly used as a marker of S-phase of the cell cycle. MiR-134 mimics decreased the expression level of PCNA proteins (Figure 2a), whereas miR-134 inhibitor had no significant effect (Figure 2a).

To ascertain whether miR-134 could regulate hCMPC proliferation, the expression levels of cell cycle genes were examined by RT-PCR with GAPDH as the internal reference. We found that the over-expression of miR-134 significantly down-regulated cyclin A, cyclin B, cyclin E, cdc2, and CDK4, as well as PCNA and E2F1 (Figure 2b), while the inhibition of miR-134 increased the expression of CDK4 and E2F1 (Figure 2c). We further performed the RT-PCR experiment using 18s rRNA as internal reference and these results were confirmed (Figure S2).

In addition, the TUNEL staining test and detection of Caspase 3 expression showed that miR-134 mimics did not induce hCMPCs apoptosis (Figure S3). The results indicate that the effect of miR-134 mimics in hCMPCs was not related to cell apoptosis. Taken together, miR-134 regulates the proliferation of hCMPCs *in vitro*.

### 2.2. miR-134 Is Not Required for the Terminal Differentiation of hCMPC into Cardiomyocytes in Our Model

**Figure 3:**
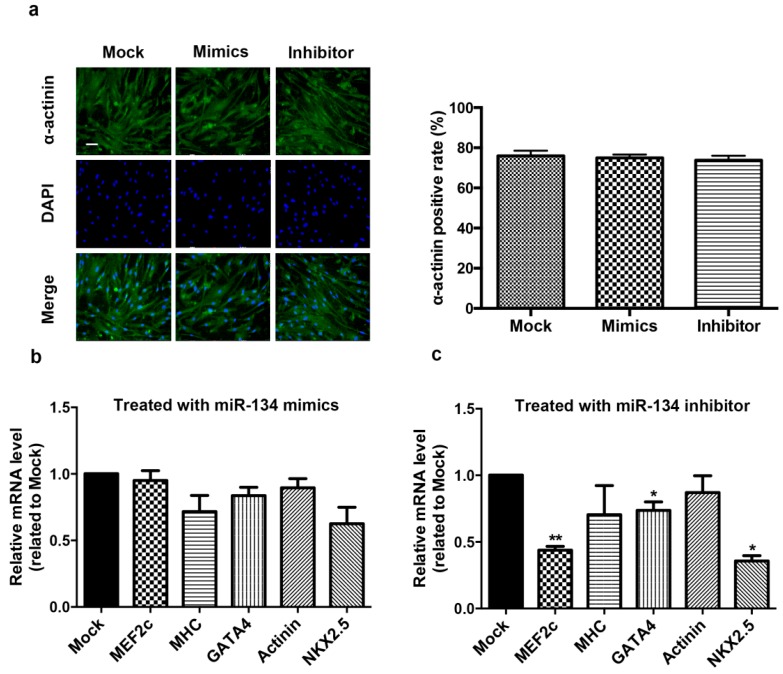
Effect of miR-134 on hCMPC differentiation to cardiomyocytes. (**a**) Representative image of differentiating hCMPCs with modulation of miR-134 or mock. Cells were stained with DAPI and α-actinin. Bar = 75 μm. Data collected from the different groups transfected with mock, miR-134 mimics or inhibitor; (**b**) Relative expression of cardiac genes in differentiating hCMPCs transfected with miR-134 mimics; (**c**) The same markers were detected in hCMPCs with the inhibition of miR-134. GAPDH levels were used to normalize the gene-specific expression levels. * *p* < 0.05, ** *p* < 0.01. Data were from six independent experiments.

hCMPCs have the capacity for unlimited growth and to differentiate into cardiomyocytes *in vitro*. We examined the expression level of miR-134 during hCMPC differentiation (Figure S4). The result showed that miR-134 level was not significantly changed in the process. To determine whether the presence of miR-134 is a prerequisite for hCMPC differentiation, we modulated miR-134 expression in hCMPCs and quantified the degree of differentiation by staining for sarcomeric α-actinin. After two weeks of differentiation, we found that neither the miR-134 mimics nor the inhibitor changed the ratio of α-actinin positive cardiomyocytes compared with the control (Figure 3a). Furthermore, the mRNA expression levels of sarcomeric proteins, which are myocardial-specific molecular markers, were also detected. miR-134 over-expression did not change the transcriptional levels of MEF2C, β-MHC, GATA4, actinin, or Nkx2.5, but its inhibition decreased the expression of MEF2C, GATA4, and Nkx2.5 (Figure 3b,c). These results indicate that miR-134 may affect the early differentiation of hCMPCs while does not determine the terminal differentiation into cardiomyocytes in our model, suggesting that miR-134 is not a prerequisite in this process.

### 2.3. Meis2 Is Identified as the Target of miR-134

**Figure 4:**
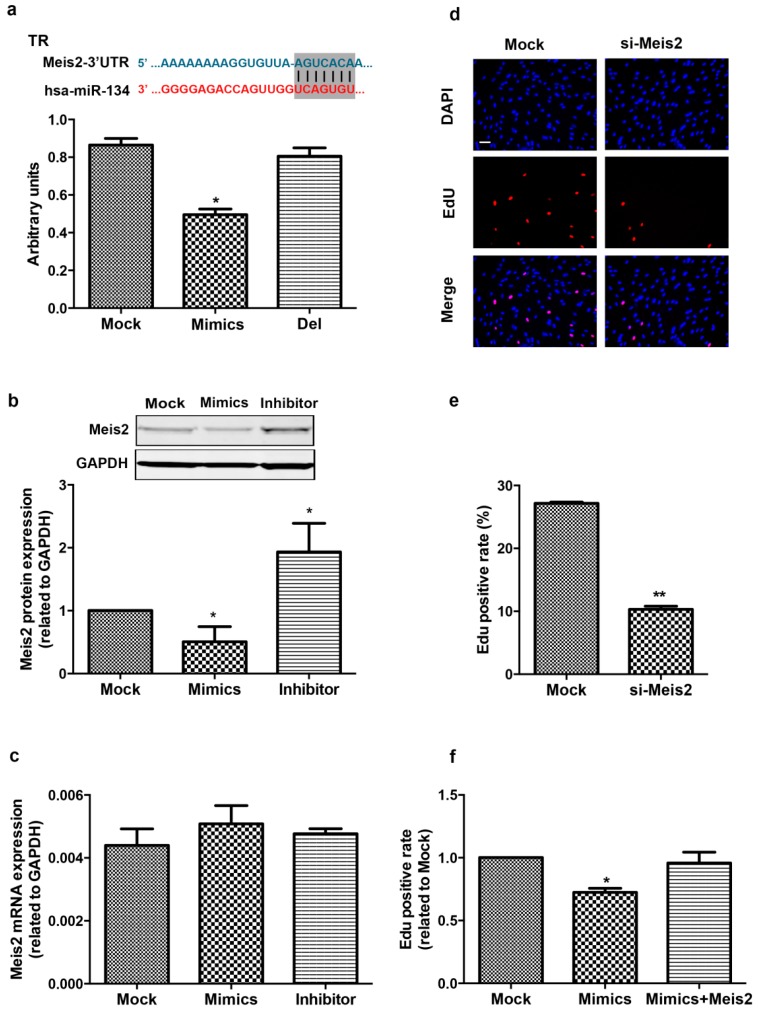
*Meis2* is the target of miR-134. (**a**) miR-134 directly targets the binding site in *Meis2*, as predicted by bioinformatics, and is highlighted in black. The blue words indicate the 3′UTR sequence of human *Meis2*; The red words indicate the sequence of miR-134. The relative luciferase activity is shown under various conditions using the 3′UTR target sequence of human *Meis2*; Abbreviations: TR, target region; Del: deletion region of TR. Data were from four independent experiments; (**b**) Meis2 protein was significantly changed in hCMPCs after treatment with miR-134 mimics or inhibitor. Data were from three independent experiments; (**c**) mRNA levels of *Meis2* were not altered in different groups. Data were from three independent experiments; (**d**,**e**) Representative images and statistical data of *Meis2* interference. *Meis2* siRNA reduced EdU incorporation. Bar = 75 μm. Data were from five independent experiments; (**f**) Overexpression of *Meis2* rescued the proliferative effect of miR-134 mimics in hCMPCs. Data were from five independent experiments. * *p* < 0.05, ** *p* < 0.01.

To explore the mechanisms of miR-134 in the regulation of hCMPC proliferation, we screened the candidate targets of miR-134 that have been reported to be involved in cell proliferation and/or differentiation, using the bioinformatic approach of TargetScan. TargetScan predicted myeloid ecotropic insertion site 2 (*Meis2*) as a potential target, and we then performed luciferase reporter assays to test whether miR-134 directly binds to *Meis2*. The 3′UTR of *Meis2* containing the putative miR-134 binding site was cloned downstream of a luciferase reporter gene and cotransfected with either miR-134 mimics or an unrelated scramble. As indicated in Figure 4a, the cotransfection of miR-134 mimics with the 3′UTR luciferase reporter of *Meis2* reduced the luciferase activity. Mutation of the binding site within the *Meis2* 3′UTR abolished the repression, revealing that miR-134 directly binds to the 3′UTR of *Meis2*.

The mRNA and protein level of *Meis2* were detected in hCMPCs after treatment with miR-134 mimics or inhibitor to conform whether miR-134 could endogenously regulate *Meis2*. We found that miR-134 mimics decreased the protein level of Meis2 and miR-134 inhibitor increased it (Figure 4b). RT-PCR showed that the mRNA level of *Meis2* was not altered with transfection of miR-134 mimics or inhibitor, respectively, compared with the scramble (Figure 4c), suggesting that the regulation of miR-134 is posttranscriptional modification without influencing the transcriptional expression of *Meis2*. Collectively, *Meis2* is the direct and specific target gene of miR-134.

### 2.4. Meis2 Over-Expression Rescues the Effect of miR-134 on hCMPC Proliferation

To gain further insights into the molecular mechanism for the proliferation effect of miR-134, we examined the DNA synthesis of *Meis2* interference on hCMPCs. Inhibition of *Meis2* in hCMPCs reduced EdU incorporation, which is consistent with miR-134 over-expression (Figure S5 and Figure 4d). Considering miR-134 acts as a negative regulator of *Meis2*, we also examined whether *Meis2* over-expression could rescue the proliferative effect of miR-134 on hCMPCs. The result showed that the over-expression of *Meis2* abolished the reduction of miR-134-mediated hCMPC proliferation and rescued the effects of miR-134 on cell cycle gene expression (Figure 4e and Figure S6). These data suggest that miR-134 regulates hCMPCs proliferation by targeting *Meis2*.

## 3. Discussion

miRNAs manage the protein dosage of important cellular pathway genes through post-transcriptional repression and provide fine regulation of many cardiogenic processes [11,12,13]. Here, we found that miR-134 could regulate hCMPC proliferation and target the transcription factor *Meis2*, which is involved in various developmental programs [14]. Over-expression of miR-134 decreased the proliferation of hCMPCs, and *Meis2* rescues the phenotype. This study provides *in vitro* evidence that miR-134 regulates cardiac progenitor cell biology, suggesting a potential role of miR-134 in cardiogenesis.

The behavior of cells, both progenitor cells and differentiated cardiomyocytes, is strictly regulated in a spatiotemporal fashion in cardiac morphogenesis to ensure that the heart attains its correct size, structure and function [1]. hCMPCs are pluripotent cells isolated from human fetal heart tissue and could be differentiated electrophysiologically and immunologically into cardiomyocytes with great efficiency *in vitro* [5,15]. hCMPC-derived cardiomyocytes displayed excitation-contraction coupling which involves L-type calcium channel activity. They also respond to β-adrenergic stimulation. In addition to myogenic differentiation, CMPCs are able to form vascular sprouts when cultured on Matrigel, showing their progenitor potential [15]. There are complex molecular networks underlying hCMPC proliferation and differentiation in which miRNAs play fascinating roles [16,17,18]. Our previous work showed that miR-134 was one of the most downregulated miRNAs in TOF patients, which suggests its possible involvement in cardiogenesis [10]. In the present study, over-expression of miR-134 decreased EdU and Ki-67 positive hCMPCs, demonstrating the inhibition of DNA and ribosomal RNA synthesis. The downregulation of miR-134 displayed a reverse effect, implying that miR-134 negatively regulates the proliferation of hCMPCs.

The precise progression of cell proliferation requires organized expression and activation or inactivation of both positive and negative regulators of the cell cycle [19,20]. We detected the expression of several cell cycle regulators in hCMPCs after treatment with miR-134 mimics or inhibitor. Though over-expression of miR-134 led to the upregulation of most cell cycle genes, only CDK4 and E2F1 changed in hCMPCs with the inhibition of miR-134. The cyclin dependent kinases (CDKs) are positive regulators of cell cycle progression [21]. Over-expression of E2F transcription factors has been reported to promote cardiomyocyte proliferation [22]. The activation of CDKs requires direct interaction with their specific cyclin partner(s) at specific phases of the cell cycle [21]. Both CDK4 and E2F1 are involved in G1/S-phase transition [23]. We assume that the inhibition of miR-134 promotes the proliferation of hCMPCs though G1/S transition and undergoing DNA synthesis. However, more studies are needed to illustrate the mechanism.

miR-134 is brain-enriched and was first reported to be involved in synaptic development, maturation and plasticity [24]. As an inducer of pluripotent stem cell differentiation, miR-134 can enhance the differentiation of mouse embryonic stem cells (mESCs) to ectodermal lineages through the regulation of multiple mRNAs [25]. It has been shown that miR-134 plays a role in the development of the embryonic stem cell-orientated differentiation to the central nervous system by suppression of Nanong [26]. However, little is known about the function of miR-134 in cardiogenic processes, especially in cardiomyocyte differentiation and in cardiogenesis. We found that modulation of miR-134 expression did not change the degree of hCMPC differentiation into cardiomyocytes in our model, suggesting that miR-134 is not required in this process. Considering the powerful effect of miR-134 in mESC differentiation, we think that the distinct biological characteristics of the two cells should account for the difference. In addition, cardiac progenitor cells arise in the mesodermal layer, unlike mESCs and neurons.

*Meis2* is a member of the three amino-acid loop extension (TALE) family of homeo-domain-containing transcription factors, which function as regulators of cell proliferation and differentiation during embryonic development [14,27]. *Meis2* has been associated with cardiac septal defects and cleft palate as well as intellectual disability [28]. Recently, an essential role of *Meis2* in the transcriptional control of M-phase cell cycle progression was presented in neuroblastoma cells [29]. It was shown that the depletion of *Meis2* in neuroblastoma cells leads to M-phase arrest and mitotic catastrophe. *Meis2* was identified as a transcription activator that functions as a master regulator of cell cycle gene expression. In this study, we found that miR-134 negatively regulates the proliferation of hCMPCs by directly targeting *Meis2*, and *Meis2* interference promotes hCMPC proliferation. These findings link miR-134 to a development-related gene that is also involved in the control of the cell cycle, implying a potential role of miR-134 in heart development.

## 4. Experimental Section

### 4.1. hCMPC Isolation and Culture

This study was approved by the ethical committees of Tongji University School of Medicine. hCMPCs were acquired and characterized as in our previous study [5]. Briefly, fetal hearts were collected and then treated with collagenase. The cardiomyocyte progenitor cells were isolated using the Sca-1 antibody (eBioscience, San Diego, CA, USA) by magnetic cell sorting (MACS, Miltenyl Biotec, Hercules, CA, USA). hCMPC were cultured in growth medium (22% EGM-2, 66% M199, 10% FBS, 1% penicillin/streptomycin, and 1% MEM nonessential amino acids) (Gibco, Carlsbad, CA, USA) that was changed every other day and passaged when they grew to 80%~90% confluence.

### 4.2. miRNA Transfection

hsa-miR-134 mimics (double-stranded, sense: 5′-UGUGACUGGUUGACCAGAGGGG-3′, anti-sense: 5′-CCUCUGGUCAACCAGUCACAUU-3′), inhibitor (5′-CCCCUCUGGUCAACCAGUCACA-3′) and negative scramble (mock) were obtained from Genepharm (Shanghai, China). The oligonucleotides were transfected into hCMPCs using a siPORT NeoFX transfection agent (AM4511, Ambion, Carlsbad, CA, USA) following the manufacturer’s transfection protocol. Briefly, the transfection agent was diluted in Opti-MEM medium, incubated for 10 min at room temperature, added with RNA molecules, and dispensed into freshly-trypsinized cells. The cells were then cultured as usual, and the transfection medium was replaced after 12 h. The expression level of miR-134 was confirmed by RT-PCR after 48 h.

### 4.3. hCMPC Proliferation Determination

Cell proliferation was evaluated using the Click-IT EdU 555 Imaging kit (C10338, Life Technologies, Carlsbad, CA, USA) and Ki-67 immunostaining. hCMPCs were transfected with miR-134 mimics (50 nM) or inhibitor (50 nM), respectively for 48 h. For the EdU incorporation assay, 10 μM EdU was added to the hCMPCs, and the cells were then incubated for 12 h. The hCMPCs were fixed with 4% formaldehyde and treated with 0.5% Triton X-100 for 15 min at room temperature. After washing with PBS, the reaction cocktail was added to each well. The hCMPCs were stained with DAPI and visualized under a Live Cell Imaging System (Leica AF 7000, Leica, Germany). For the Ki-67 immunostaining analysis, the hCMPCs were fixed with 4% paraformaldehyde and permeabilized with 0.1% Triton X-100 at room temperature. The anti-Ki-67 (ab16667, abcam, Cambridge, MA, USA) was incubated overnight. The secondary antibody was fluorescein-conjugated goat anti-rabbit IgG (A-21428, Life Technologies). The proliferation of hCMPCs was also detected by PCNA expression.

### 4.4. Cell Viability and Apoptosis Assay

Cell viability was determined using the Cell Counting Kit-8 (CK04, Dojindo Laboratories, Tokyo, Japan). Briefly, hCMPCs were seeded into a 96-well plate at a density of 1 × 10^4^ cells per well. After transfection for 48 h, CCK-8 solution was added and incubated for 4 h. The absorbance was measured at 450 nm using the Spectra Max5 (Molecular Devices, Sunnyvale, CA, USA). Cell apoptosis was detected by the TUNEL assay (C1089, Beyotime, Shanghai, China) according to the manufacturer’s instructions.

### 4.5. Induction and Quantification of hCMPC Differentiation

To induce the differentiation of hCMPCs into cardiomyocytes, hCMPCs were seeded in a 6-well plate and incubated overnight. The next morning, the growth medium was changed to differentiation medium and 5 µM 5-azacytidine (A2385, Sigma, St. Louis, MO, USA) was added for three days. The cells were transfected with miR-134 mimics or inhibitor or scramble control as described above at four days after the start of the differentiation. Then, hCMPCs were treated with transforming growth factor (TGF)-β (1 ng/mL, 100-21c, PeproTech, Rocky Hill, NJ, USA) for 14 days, as a previous study described [5,15]. To quantify the degree of differentiation, the expression of cardiac genes was tested by RT-PCR. α-Actinin (sarcomeric) (A7811, Sigma) was also used following the manufacturer’s protocol. The cells were photographed and analyzed with the Live Cell Imaging System (Leica AF 7000, Leica).

### 4.6. Luciferase Experiment

The region of *Meis2* 3′UTR was amplified from human genomic DNA by PCR using specific primers (F: 5′-GCTCTAGACCTCTCTATTTTCAGGTTTG-3′; R: 5′-GCTCTAGAAGCCTAACAAAAAATCAAAA-3′). The fragment was inserted into the pmirGlo luciferase gene to construct the luciferase reporter plasmid of *Meis2*. The predicted binding site (AGTCACA) in the 3′UTR of *Meis2* was mutated for contrast. 293 cells were transfected with luciferase reporter plasmids and miR-134 mimics (50 nM) by Lipofectamine 2000 (11668-019, Invitrogen, Carlsbad, CA, USA). The luciferase activities were measured using a dual luciferase reporter assay system (E1910, Promega, Madison, WI, USA).

### 4.7. Modulation of Meis2 in hCMPCs

To construct the *Meis2* overexpression plasmid, the CDS region of human *Meis2* was subcloned into the PCDNA 3.0 vector. The transfection of the *Meis2* plasmid into hCMPCs followed the manufacturer’s instructions using Lipofectamine 2000 (11668-019, Invitrogen). *Meis2* siRNA (5′-CACCCUGGAAUGACUAUGUTT-3′ and 5′-ACAUAGUCAUUCCAGGGUGTT-3′) was purchased from GenePharma. The expression of Meis2 was measured by western blot.

### 4.8. RT-PCR

Total RNA was isolated using the mirVana™ miRNA Isolation kit (Ambion Inc., Carlsbad, CA, USA). For the quantitative detection of miRNA expression, the miScript Reverse Transcription (RT) Kit (Qiagen, Hilden, Germany) and miScript SYBR Green PCR Kit (Qiagen) were used. For the absolute quantification for miR-134, the standard oligo was ordered from GenePharma (Shanghai, China) and serial dilution was made for the PCR reaction. Then the standard curve method was used to calculate the copy numbers of miR-134. For the detection of mRNA levels, the reverse transcription reaction was carried out using PrimeScript RT reagent Kit (RR037A, Takara, Japan), following manufacturer’s instructions. Real-time PCR analyses were performed using specific sets of primers (Table S1) and SYBR Green mix (4367659, Applied Biosystems Life Technologies, Carlsbad, CA, USA). All RT-PCR reactions, including no-template controls and no reverse transcription controls, were performed in triplicate. The expression level of miR-134 and other target genes was normalized to internal reference genes, GAPDH and 18s RNA. The 2^−ΔΔ*C*t^ method was used to calculate the relative levels of target mRNA, and GAPDH and 18s RNA were employed as internal controls.

### 4.9. Western Blot

Total protein of hCMPCs was harvested with RIPA buffer (P0013, Beyotime, Shanghai, China) containing protease inhibitor cocktail (04693132001, Roche, Switzerland). Proteins were separated on NuPAGE 10% Bis-Tris Gel (NP0315BOX, Invitrogen) and then transferred to PVDF membrane. Antigens were detected using the following primary antibodies at 4 °C overnight: anti-*Meis2* (ab73164, abcam, Cambridge, MA, USA), anti-caspase 3 (9662, Cell Signaling Technology, USA), anti-PCNA (ab29, abcam, USA) and anti-GAPDH (Cell Signaling, Danvers, MA, USA), followed by incubation with Rabbit anti-sheep IgG (H + L) (072-07-23-06, KPL, Gaithersburg, MD, USA) and goat anti-mouse IgG (H + L) (072-02-18-06, KPL, Gaithersburg, MD, USA) fluorescent secondary antibodies. The signal was visualized with Odyssey (Li-Cor, Lincoln, NE, USA). The intensity of the immunoblots was analyzed using Quantity One imaging software (Bio-Rad, Hercules, CA, USA).

### 4.10. Statistical Analysis

Data are displayed as the mean ± SE. An independent-sample *t*-test or one-way ANOVA was used to assess the differences among the experimental groups. *p* values <0.05 were considered to indicate statistical significance.

## 5. Conclusions

In summary, our findings present roles of miR-134 in the regulation of cardiac progenitor cell biology. The over-expression of miR-134 decreased the proliferation of hCMPCs, while the inhibition of miR-134 induced the opposite effect. Modulation of miR-134 altered the transcriptional level of cell cycle genes. *Meis2*, a transcription factor that is involved in cell cycle progression and various development processes, was identified as the target of miR-134. This study provides clues for the study of physiology and pathology of cardiogenesis.

## Supplementary Materials

Supplementary materials can be found at http://www.mdpi.com/1422-0067/16/10/25199/s1.
