# Supplementary material for: miR-134 Modulates the Proliferation of Human Cardiomyocyte Progenitor Cells by Targeting Meis2
Source: Int J Mol Sci. 2015 Oct 23;16(10):25199–213. doi: 10.3390/ijms161025199 (PMC4632798; doi:10.3390/ijms161025199)
Supplement: Supplementary file 1 [file ijms-16-25199-s001.pdf]

Supplementary Information

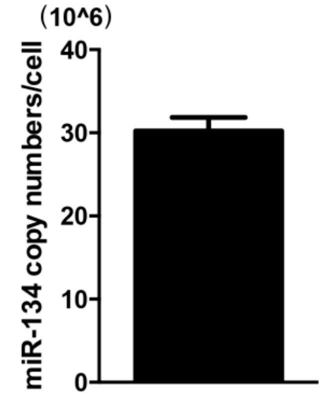

Figure S1. The expression level of miR-134 in proliferating hCMPCs. Data were from 3 independent experiments.

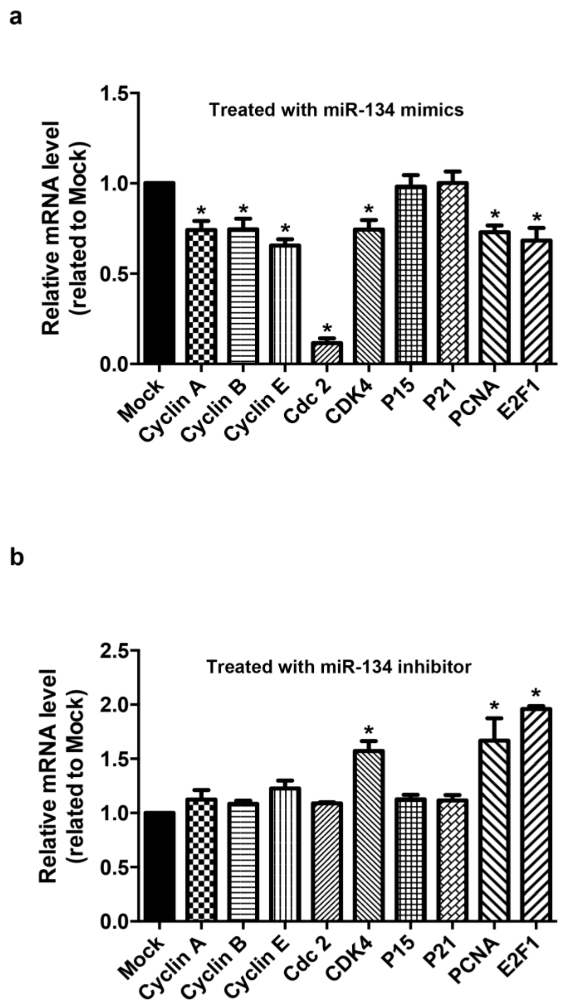

Figure S2. Modulation of miR-134 in hCMPCs changes the expression level of cell cycle genes. (**a**) Relative expression of cell cycle genes in hCMPCs treated with miR-134 mimics; (**b**) Cell cycle genes were examined in hCMPCs with the inhibition of miR-134. 18s RNA was used as the internal reference. * $p < 0.05$, Data were from 3 independent experiments.

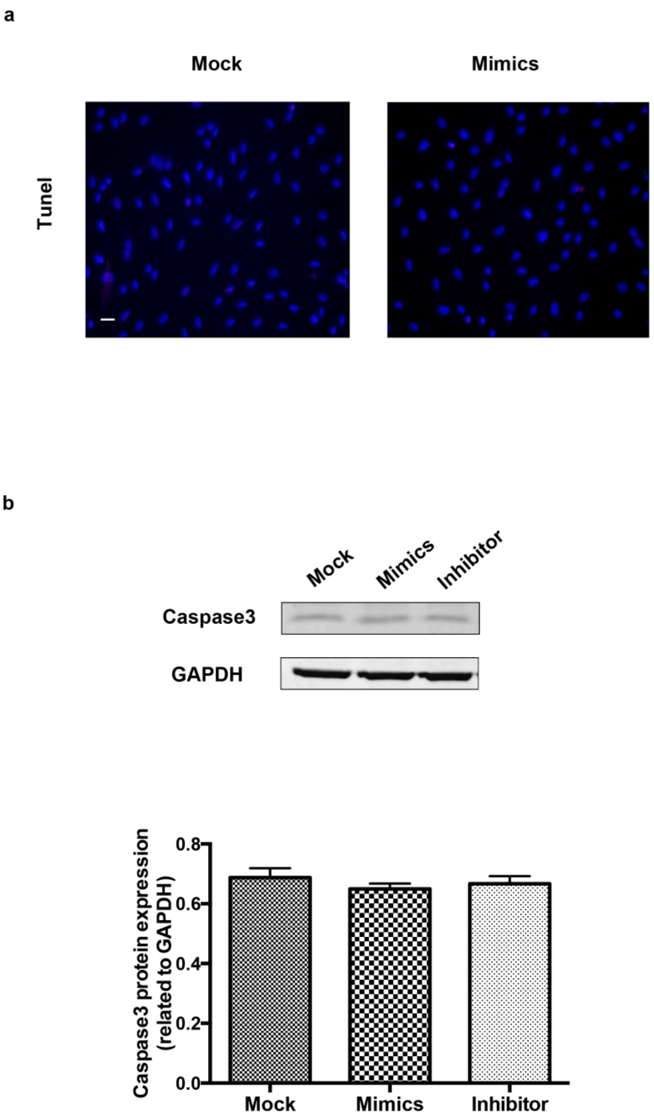

Figure S3. miR-134 does not cause hCMPCs apoptosis. (**a**) TUNEL staining assay for cell apoptosis detection; (**b**) Expression of Caspase 3 in hCMPCs transfected with miR-134 mimics or inhibitor. miR-134 mimics or inhibitor did not induce hCMPCs apoptosis. Bar = 75 μm. Data were from 3 independent experiments.

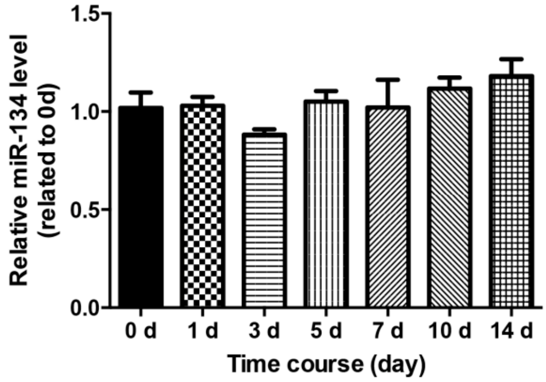

Figure S4. The expression level of miR-134 remains unchanged during hCMPCs differentiation. Data were from 3 independent experiments.

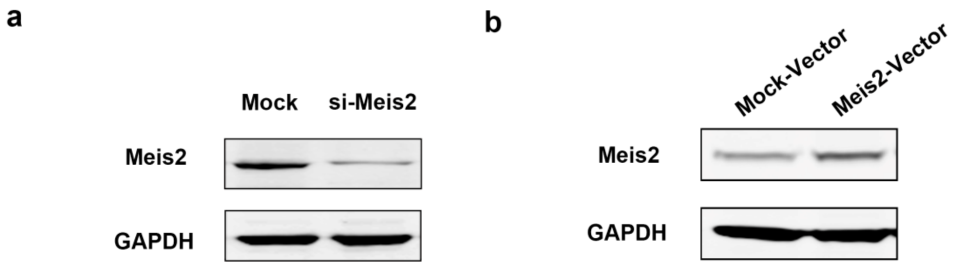

Figure S5. Expression of the Meis2 protein is altered under different treatments. The Meis2 protein level was altered in hCMPCs that were treated with si-Meis2 (**a**) or Meis2 overexpression vector (**b**).

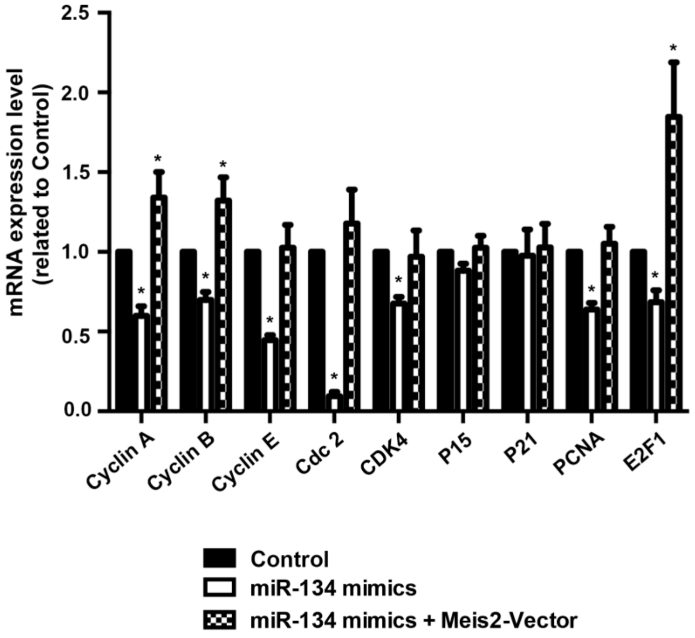

Figure S6. Over-expression of Meis2 prevents the effect of miR-134 on cell cycle genes. * $p < 0.05$, Data were from 3 independent experiments.

Table S1. Primer sequences for gene expression detection.

Genes	Primer Sequences
Meis2	5′-GTGAGCCAAGGAGCAGCATA-3′<br>5′-ACATGTAGTGCCATTGCCCA-3′
Cyclin A	5′-AACTTCAGCTTGTGGGCACT-3′<br>5′-AAACTCTGCTACTTCTGGGGG-3′
Cyclin B	5′-TGCAGCACCTGGCTAAGAAT-3′<br>5′-TAGCATGCTTCGATGTGGCA-3′
Cyclin E	5′-AAAGTTGCACCAGTTTGCGT-3′<br>5′-TCAGGGGACTTAAACGCCAC-3′
Cdc2	5′-CTTTCTTTCGCGCTCTAGCC-3′<br>5′-AATCGGGTAGCCCGTAGACT-3′
CDK4	5′-GCGTGAGGGTCTCCCTTGAT-3′<br>5′-ACCGACACCAATTTCAGCCA-3′
P15	5′-ACTAGTGGAGAAGGTGCGAC-3′<br>5′-GCCCATCATCATGACCTGGA-3′
P21	5′-AGCTGCCGAAGTCAGTTCCTT-3′<br>5′-GTTCTGACATGGCGCCTCCT-3′
PCNA	5′-GGCTCTAGCCTGACAAATGC-3′<br>5′-TCTAGCTGGTTTCGGCTTCAG-3′
E2F1	5′-GCCATCCAGGAAAAGGTGTGA-3′<br>5′-GTGATGTCATAGATGCGCCG-3′
MEF2C	5′-AGATACCCACAACACACCACGCGCC-3′<br>5′-ATCCTTCAGAGAGTCGCATGCGCTT-3′
GATA-4	5′-GACAATCTGGTTAGGGGAAGC-3′<br>5′-ACCAGCAGCAGCGAGGAGAT-3′
Nkx-2.5	5′-CGCCGCTCCAGTTCATAG-3′<br>5′-GGTGGAGCTGGAGAAGACAGA-3′
MHC	5′-GAAGCCCAGCACATCAAAAG-3′<br>5′-GATCACCAACAACCCCTACG-3′
Actin	5′-TCCTGATGCGCATTTTTATTC-3′<br>5′-AACACCACTGCTCTAGCCACG-3′
